# Pressure Anesthesia

**Published:** 1900-08-15

**Authors:** W. J. Higgins


### PRESSURE ANESTHESIA.

BY W. J. HIGGINS, D.D.S.

Pressure Anesthesia, for the removal of pulps, was first brought under my observation last November, when I attended a clinic where the practicability of this method was demonstrated.

Some time before the clinic, which was before the Toledo Dental Association, I had noticed something relative to pressure anesthesia in some of the journals, but nothing sufficient to induce me to make a trial of it until I had really seen the nicety of the operation. Since that time I have seen one or two articles relative to it. I hope my paper may evoke discussion that will be of benefit to myself and all present.

Since November I have used nothing but this method for anesthetizing pulps, and have found it equally available in incisors, bicuspids and molars. In every case I have met with success in removing the pulp and filling the roots immediately.

As I was admonished to make my paper brief and to the point I will not take time to elaborate any of the individual cases that have been presented to me any more than to quote one case, and then pass on to my method of procedure.

The case referred to was that of a young man of twenty-two years—four upper incisors badly decayed, but no exposed nerves—exposing nerves with as little pain as possible, applying method and adjusting four Logan crowns, filling roots and cementing on the crowns. This was one afternoon's work, and was a source of satisfaction to myself and to my patient. This is one case of many which I might mention where pressure anesthesia has served me well.

My Method of Procedure: After adjusting the rubber dam, to prevent the cocain from getting into the mouth and also to have a dry operation, I locate the exposure, or if there is no exposure, I expose the pulp as carefully as I can. Although it is not necessary to have a bleeding pulp, in cases where I can do so without much pain, I cause a slight flow of blood. I use the pure crystals of cocain (which can be procured at any drug store, two or three grains for five cents, according to the liberality of your druggist). Will say here that the last five cents' worth of cocain I procured was sufficient to anesthetize twenty-five pulps.

I take a few of the cocain crystals, and placing them on a glass slab, moisten the cocain with water enough to make a saturated solution; then taking a small pledget of cotton moisten with the cocain solution to saturation and apply to the exposure. Take vulcanite rubber of a size that will fit into the cavity easily, and with the slow pressure of a burnisher the pulp will be gradually anesthetized. The pressure must be light and gradual, or the pain will be severe. After there is no response to excessive pressure, the rubber and cotton pledget are removed and the pulp extracted. Often there is bleeding after removal of the pulp, which I quickly stanch by hydrogen dioxide, or if excessive, by iron persulphate. Drying the cavity thoroughly, seldom using anything of a sedative or antiseptic nature, I fill the roots and insert the filling or adjust the crown without fear of future annoyance.

There may be a slight tenderness in a tooth treated this way for a day or two, although the liability is not as great as a tooth treated with arsenic.

DISCUSSION.

Discussion opened by Dr. B. H. Lee, Grand Rapids. The subject of pressure anesthesia as far as I know it is not large enough to be elaborated to any considerable extent. Last winter I read an article which appeared in one of the journals concerning this method of extracting pulps, and it so happened that a day or two after that I had a case which gave me the opportunity to try it. I followed the directions very minutely as far as I was able to do, but I must confess in this first case I almost killed my patient, or at least she said I did. I tried it again in several cases after that and succeeded in getting out pulps of the anterior teeth very successfully.

I do not believe that it is a good method to use in all cases. I have tried it in the molar teeth and succeeded in getting the tissue out of the pulp chamber, but not out of the canals, especially the smaller canals, without a great deal of pain. It seems impossible to anesthetize these fine nerve filaments in the canals. But for the anterior teeth where we have a neat exposure to apply the cocain, it is very good for a quick operation.

I think this name of pressure anesthesia will bear criticism; it is not the pressure which causes the anesthesia, but the infiltration of the cocain into the tissues. I tried pressure alone after I was informed that I was to open discussion on this paper. I had several exposed nerves and tried pressure alone but it would not anesthetize in any case—it might be that I did not continue it long enough.

Instead of using the pure cocain crystals, I used Dr. Hoff's tablets, making a saturated solution, with good results.

Dr. Sleich is the author of anethetizing by infiltration. His method is to combine pressure with the solution which is injected into the tissue. He marks out the area to be operated upon and inserts the needle filling the tissue with the liquid. It seems this is the same idea, and infiltration is the proper name for it.

I stop the bleeding with peroxide of hydrogen. If pure and obtained from a reliable source I do not believe you need any better antiseptic.

The essayist says he does not use any other method for anesthetizing pulps. I do not believe from my own experience that this method is the best thing to use in all cases. Only once in a while do I find conditions where I can use this method successfully. I still cling to the arsenic in difficult locations, and especially in the molar teeth.

Dr. Stofflet: About Christmas time a man had a pulp taken out with pressure anesthesia. He came to me about two months ago to have a molar tooth extracted. On examining the teeth I noticed that the second bicuspid on the left side had been taken out, or at least it had been broken off. I said to him, "You have a badly decayed root there," and he replied, "No; I had a pulp taken out with pressure anesthesia, and the tooth dropped out about three months ago." There was never any soreness, and I wondered what caused that tooth to come out, and I would like to inquire if any one ever heard of a like case, and if the cocain could cause the tooth to drop out. He said he picked out several small pieces of bone.

Dr. Essig: I would like to ask if it is just as rapid to remove a pulp by the use of the hypodermic injection as with pressure anesthesia? My experience has been that I can remove very rapidly with the hypodermic, using a saturated solution of cocain, injecting into the pulp with scarcely any pain at all; the pain may be severe just for an instant. I think it is more successful than the pressure method. I have tried both.

Dr. Hines: I have used this method of injecting the pulp with cocain combined with the pressure method, and I have found that in the anterior teeth it works admirably. My idea of using the pressure method is to first anesthetize the outer surface of pulp with it, and then inject with the needle. I use concentrated solution of cocain with vulcanite rubber and slight pressure at first; then by increasing the pressure I can anesthetize sufficiently to permit painless introduction of the needle to thoroughly anesthetize the pulp. I get much better results by combining the two methods. It is impracticable to get good results in some teeth. I have not practised filling roots immediately after extraction of pulp; I treat for a day or two, and then fill, on account of serum in the canals.

Dr. Siddall: Pressure anesthesia I have used for seven or eight years at least. I began at first using cocain with hypodermic needle. I use the following method. I take a broken needle and grind it squarely off, making it about one-sixteenth of an inch long, and by using a little rubber washer over the base of the needle I am able to accomplish the same as with the vulcanite. The point of the needle is introduced into any slight exposure and the contents of syringe forced into the canal. The objection to this method is that the rubber washer closes the roots completely, and the sudden injection of the fluid is very painful. I began this method by using carbolic acid, and later cocain; since then I have used the rubber vulcanite in place of the hypodermic syringe. It is practicable to produce anesthesia by this method in any tooth in the mouth. In the use of the hypodermic we are obliged to have cases that are favorable. Ordinarily I introduce crystals of cocain, with slight amount of moisture, and cover with vulcanite. The last gentleman said he used gentle pressure; I do not want to do so. I use great pressure, and am not long about it either. I want to have my patient where he can not get away from me. I have a broad burnisher, an amalgam plugger, so-called. I get it in there so quick the patients give a little jump, and I ask them if it hurts. They say no. I then ask them what they jump for, and they do not know. It is so quick that it is as near painless as anything can be. I used carbolic acid just the same way; it cooks the tissue, and I get the whole pulp out slick and clean. If I do not get it out clean I use sulphuric acid, plenty of it and full strength. In teeth of good access I can get the pulps out almost every time, and do it quickly. Within an hour after the patient comes into my office I can have the pulp out and root filled—yes, within forty minutes, but I do not do it in summer. Occasionally with such treatment the tooth will be sore for several days and sometimes for several weeks. Of course, if there is lots of trouble the patient goes to somebody else and complains about me, but I have kept track of enough cases to know that the results are very satisfactory. I have my routine treatment, which is just as I have described it, and I fill with a dressing of eucalyptus and do not use enough to be forced through end of root. That is a condition where I think we are liable to make trouble. If there is a septic condition of the pulp and you produce pressure for anesthetic purposes, there is a possibility that the septic material will be forced through the apex of the root.

Dr. Oakman: I think, Dr. Siddall, if you use dilute sulphuric acid it would be more active.

Dr. Siddall: It is diluted enough by the fluids of the pulp canal.

Dr. Oakman: In my opinion, to use a hypodermic needle without first applying cocain topically is bad practice. By anesthetizing the pulp superficially we can introduce the needle without any pain whatsoever in the operation. I have followed that method for possibly four years, not so much with the pressure at first, but simply apply cocain to the pulp before injecting. During the last year I have used the pressure method; by so doing it is possible to anesthetize the pulp to the apex of the root.

DR. ROOT: I was just rising to ask how many dentists here have ever had pressure anesthesia tried upon their teeth? I should like to know how far this operation can be done painlessly. The old method answers my purpose very nicely. If you want to lose your patients, try pressure anesthesia. I don't think it is possible to use this method painlessly. I have been through the operation within the last few weeks, and I tell you it is extremely painful; and not only that, it is liable to hurt after the pulp is out. Great care should be exercised in its use.

One gentleman said: "You want to get patient so he can not get away from you." Now, to my mind, that is wrong. They should not feel as if they were in a vise, but they should be quiet and say they feel no pain at all. You can do this if you take the old-fashioned way, with arsenic, keeping the treatment in for a few days. It is along the same line as taking a peg and driving it up into canal, etc. It is the same principle, and we called it painless extraction of the pulp. To-day we do it by pressure anesthesia.

DR. HINES: I have never had it tried on myself, but I have tried it upon my wife—and if there is any one under the sun who would kick it is your wife.

DR. CORNS: We hear the different gentlemen telling us that they anesthetize the pulp with cocain. If so, I do not see the necessity of applying pressure. It can be anesthetized without the pressure. Or, why not use pressure alone? I am not sure that I understand, and I wish some one would explain the method to me. Do you first apply the cocain on a piece of cotton and put the rubber over that?

DR. SWEETNAM: I have heard this paper discussed with a good deal of satisfaction. No one has as yet mentioned the method I most like, and if it going to do any good I will be glad to give that method.

In the first place I use arsenic in some combination to devitalize the pulp, if possible; but I find in applying arsenic that it does not have the same affinity in different cases and that it requires different lengths of time to destroy the pulp. We think sometimes we have devitalized, when on opening we find we have not. Perhaps the body of the pulp is entirely destroyed, but when we come into the smaller ends of roots—the anterior roots of lower molars, for example—we find a considerable degree of sensitiveness. In these cases I prefer to open this up by the use of a very fine wire with the very smallest amount of bibulous paper wrapped around it. If you do this bunglingly you will have something which will not go into canal, but if done correctly the work will give great gratification. After dipping this in the carbolic acid or creosote so as to thoroughly destroy all germs, then get out the pulp by means of broaches and drills. By doing it in this way I cause the least pain possible, and that is the way I should prefer to have it done for myself.

Dr. Oakman: The question was asked, "Why use pressure and then the hypodermic?" It is simply to relieve the pain which comes from the insertion of the needle into the pulp. That is the only reason I use it.

Dr. Corns: Why use pressure after getting anesthesia of pulp, what more do you need?

Dr. Oakman: That is to cause it to anesthetize deeply. The gentleman spoke about arsenic. I think it has its place. I don't believe in adhering to any one thing for all cases; there is no one thing which is successful in every instance, and I think all who have practiced dentistry know that from experience. If the pulp is highly congested and you want to remove it, and it is to be devitalized and you do not wish to apply a sedative, then cocain with pressure is just the thing; there is nothing better. Ninety-nine times out of a hundred the patient will experience no pain. It is not necessary to use arsenic where you use cocain, except where the canals are fine.

If you inject cocain into a pulp canal it will penetrate to the apical foramen; you can not produce any better effect than this with arsenic. Then, by the use of drills, or any of the ways suggested last evening, there are various ways of removing pulp; it can be taken out with very little pain. I have done it successfully for some time and do not cause any pain to speak of.

Dr. Blackmarr: I am surprised that Dr. Hines should have tried this on his wife; the most of us would have tried it upon our mother-in-law. I have been away from home about six weeks, and I have really forgotten whether pressure produces anesthesia or not.

Dr. Higgins, closing the discussion: As to arsenic I had a little experience in a tooth of my own, which I had to lose. It was a first lower right bicuspid. I lost it about six months ago. It was a fairly good tooth, it was treated with arsenic to destroy pulp, cleaned out and filled. For months afterward I suffered continual pain, finally I had to lose it and I have it with me here to day. I usually bring along specimens every time I come to the meeting of the association, and I will show this to the gentlemen after the meeting is over. There is absorption of root half way up, and there was a very large abscess on the root. I asked several gentlemen what they thought was the cause of this absorption and they all said arsenic, and I did not tell them that arsenic had been used. I have seen other cases of similar nature caused by arsenic. As to name of pressure anesthesia, I will ask the gentlemen when they go home to try this pressure anesthesia, and then they will know where the name comes from; if they try cocain without the pressure they will find there is no anesthesia produced; that is, there is no permeation of the pulp. Electricity, of course, is used to bring about this result, but without electricity or pressure cocain for anesthetizing pulps will not be satisfactory. Pressure anesthesia is rightly named.

In regard to using arsenic, I think that Dr. Sweetnam brought out a very good point. After using arsenic first in many cases we do not have devitalization of the pulp. Dr. Lee said cocain would not permeate the pulp sufficiently to anesthetize the pulp to the end of the root. Cocain is much better for this purpose than arsenic, it will anesthetize to end of root. If you use arsenic first and cocain afterward you will be convinced.

In reference to Dr. Root's statement, all I will ask him to do is to try the method as given and next year let him come here and tell us his experience; if he follows directions he is sure to succeed.

Dr. Crouse then spoke on Dental Protective Association. Following same motion was made to appoint a committee to express sentiment of the Association in reference to same.

Dr. Wm. E. Harper, of Chicago, then read a paper on "The Preparation of Cavities for Contour Fillings."

